# Supplementary material for: A tool for the cheap and rapid screening of SARS-CoV-2 variants of concern (VoCs) by Sanger sequencing
Source: Microbiol Spectr. 2023 Sep 7;11(5):e05064-22. doi: 10.1128/spectrum.05064-22 (PMC10586709; doi:10.1128/spectrum.05064-22)
Supplement: Supplemental Information — Fig S1: Chromatograms that show ~ 80bp long genetic profiles (between OMI6 to N501Y) of four samples. Table S1: Diagnostic Mutations of samples used for validation respect to the Wuhan reference sequence (NC_045512.2). [file spectrum.05064-22-s0001.pdf]

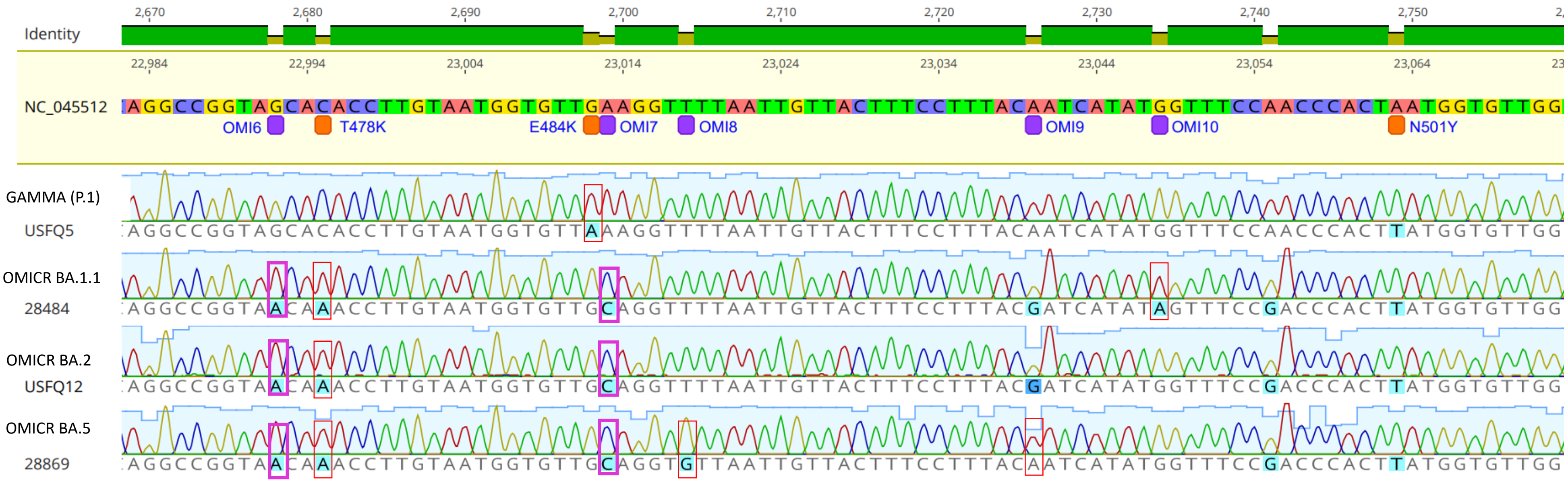


**Fig S1**. Chromatograms that show genetic profiles (between OMI6 to N501Y ~80bp) of four samples. Diagnostic mutations for each variant are highlighted with red squares, while Omicron-defining mutations are highlighted in purple ones.

| REF. WUHAN (NC_045512.2) | | C | A | T | C | A | G | A | T | G | C | G | A | T | A | G | A | C | A | C | C | C | GenBank ID | WGS Accesion code | Country | Host | Collection Date | Isolation Source |
|---|---|---|---|---|---|---|---|---|---|---|---|---|---|---|---|---|---|---|---|---|---|---|---|---|---|---|---|---|
| Isolate | Lineage | T19R<br>C21618G | D80A<br>A21801C | OMI1<br>T22673C | OMI2<br>C22674T | OMI3<br>A22688G | OMI4<br>G22775A | K417T<br>A22812C | OMI5<br>T22917G | OMI6<br>G22992A | T478K<br>C22995A | E484K<br>G23012A | OMI7<br>A23013C | OMI8<br>T23018G | OMI9<br>A23040G | OMI10<br>G23048A | N501Y<br>A23063T | OMI11<br>C23202A | S12<br>A23403G | T716I<br>C23709T | OMI12<br>C27889T | OMI13<br>C28724T | | | | | | |
| USFQ6 | WUHAN (NC_045512.2) | C | A | T | C | A | G | A | T | G | C | G | A | T | A | G | A | C | A | C | C | C | OQ978576<br>OR054013<br>OQ978577 | EPI_ISL_486843 | Ecuador | Homo sapiens | 2020-06-27 | Nasopharyngeal swab |
| USFQ1 | ALPHA (B.1.1.7) | - | - | - | - | - | - | - | - | - | - | - | - | - | - | - | T | - | G | T | - | - | OQ978564<br>OQ978563<br>OR141938 | EPI_ISL_2361471 | Ecuador | Homo sapiens | 2021-10-05 | Nasopharyngeal swab |
| USFQ3 | ALPHA (B.1.1.7) | - | - | - | - | - | - | - | - | - | - | - | - | - | - | - | T | - | G | T | - | - | OQ978568<br>OQ978569<br>OR141939 | EPI_ISL_2100429 | Ecuador | Homo sapiens | 2021-04-30 | Nasopharyngeal swab |
| USFQ2 | GAMMA (P.1) | - | - | - | - | - | - | C | - | - | - | A | - | - | - | - | T | - | G | - | - | - | OQ978565<br>OQ978566<br>OQ978567 | EPI_ISL_2361481 | Ecuador | Homo sapiens | 2021-05-20 | Nasopharyngeal swab |
| USFQ5 | GAMMA (P.1) | - | - | - | - | - | - | C | - | - | - | A | - | - | - | - | T | - | G | - | - | - | OQ978573<br>OQ978574<br>OQ978575 | EPI_ISL_2689841 | Ecuador | Homo sapiens | 2021-06-18 | Nasopharyngeal swab |
| USFQ4 | DELTA (B.1.617.2) | G | - | - | - | - | - | - | G | - | A | - | - | - | - | - | - | - | G | - | - | - | OQ978570<br>OR083679<br>OQ978571<br>OQ978572 | EPI_ISL_4256791 | Ecuador | Homo sapiens | 2021-08-31 | Nasopharyngeal swab |
| USFQ7 | DELTA (B.1.617.2) | G | - | - | - | - | - | - | G | - | A | - | - | - | - | - | - | - | G | - | - | - | OQ978578<br>OR083680<br>OQ978579<br>OQ978580<br>OQ978581 | EPI_ISL_3506199 | Ecuador | Homo sapiens | 2021-08-04 | Nasopharyngeal swab |
| 28543 | Omicron BA.1.1 | - | - | C | T | - | - | - | - | A | A | - | C | - | G | A | T | A | G | - | - | - | OQ978594<br>OQ978595<br>OQ978596 | EPI_ISL_17708351 | Ecuador | Homo sapiens | 2022-01-14 | Nasopharyngeal swab |
| 28520 | Omicron BA.1.1 | - | - | C | T | - | - | - | - | A | A | - | C | - | G | A | T | A | G | - | - | - | OQ978597<br>OQ978598 | EPI_ISL_17708350 | Ecuador | Homo sapiens | 2022-01-14 | Nasopharyngeal swab |
| 28643 | Omicron BA.1.1 | - | - | C | T | - | - | - | - | A | A | - | C | - | G | A | T | A | G | - | - | - | OQ978599<br>OQ978600<br>OQ978601 | EPI_ISL_17708356 | Ecuador | Homo sapiens | 2022-02-17 | Nasopharyngeal swab |
| 28704 | Omicron BA.1.1 | - | - | C | T | - | - | - | - | A | A | - | C | - | G | A | T | A | G | - | - | - | OQ978604<br>OQ978602<br>OQ978603 | EPI_ISL_17708347 | Ecuador | Homo sapiens | 2022-10-03 | Nasopharyngeal swab |
| 28722 | Omicron BA.1.1 | - | - | C | T | - | - | - | - | A | A | - | C | - | G | A | T | A | G | - | - | - | OQ978607<br>OQ978606<br>OQ978605 | EPI_ISL_17708352 | Ecuador | Homo sapiens | 2022-03-17 | Nasopharyngeal swab |
| 28685 | Omicron BA.1.1 | - | - | C | T | - | - | - | - | A | A | - | C | - | G | A | T | A | G | - | - | - | OQ978611<br>OQ978610<br>OQ978608<br>OQ978609 | EPI_ISL_17708349 | Ecuador | Homo sapiens | 2022-03-02 | Nasopharyngeal swab |
| 28698 | Omicron BA.1.1 | - | - | C | T | - | - | - | - | A | A | - | C | - | G | A | T | A | G | - | - | - | OQ978615<br>OQ978614<br>OQ978612<br>OQ978613 | EPI_ISL_17708359 | Ecuador | Homo sapiens | 2022-03-07 | Nasopharyngeal swab |
| 28484 | Omicron BA.1.1 | - | - | C | T | - | - | - | - | A | A | - | C | - | G | A | T | A | G | - | - | - | OQ978617<br>OQ978618<br>OQ978619<br>OQ978620<br>OQ978616 | EPI_ISL_17708355 | Ecuador | Homo sapiens | 2022-01-10 | Nasopharyngeal swab |
| 28668 | Omicron BA.1.1 | - | - | C | T | - | - | - | - | A | A | - | C | - | G | A | T | A | G | - | - | - | OQ978624<br>OQ978623<br>OQ978621<br>OQ978622<br>OQ978625 | EPI_ISL_17708357 | Ecuador | Homo sapiens | 2022-02-25 | Nasopharyngeal swab |
| 28684 | Omicron BA.1.1 | - | - | C | T | - | - | - | - | A | A | - | C | - | G | A | T | A | G | - | - | - | OQ978629<br>OQ978628<br>OQ978626<br>OQ978627<br>OQ978630 | EPI_ISL_17708358 | Ecuador | Homo sapiens | 2022-03-02 | Nasopharyngeal swab |
| 28736 | Omicron BA.1.1 | - | - | C | T | - | - | - | - | A | A | - | C | - | G | A | T | A | G | - | - | - | OQ978634<br>OQ978633<br>OQ978631<br>OQ978632<br>OQ978635 | EPI_ISL_17708360 | Ecuador | Homo sapiens | 2022-03-22 | Nasopharyngeal swab |
| 28708 | Omicron BA.1.1 | - | - | C | T | - | - | - | - | A | A | - | C | - | G | A | T | A | G | - | - | - | OQ978639<br>OQ978638<br>OQ978636<br>OQ978637<br>OQ978640 | EPI_ISL_17708361 | Ecuador | Homo sapiens | 2022-03-14 | Nasopharyngeal swab |
| 28482 | Omicron BA.1.1 | - | - | C | T | - | - | T | - | A | A | - | C | - | G | A | T | A | G | - | - | - | OQ976989<br>OQ976990<br>OQ976991<br>OQ976992<br>OQ976988 | EPI_ISL_17708354 | Ecuador | Homo sapiens | 2022-01-10 | Nasopharyngeal swab |
| 28639 | Omicron BA.1.1 | - | - | C | T | - | - | - | - | A | A | - | C | - | G | A | T | A | G | - | - | - | OQ976987 | EPI_ISL_17708348 | Ecuador | Homo sapiens | 2022-02-16 | Nasopharyngeal swab |
| USFQ8 | Omicron BA.2 | T | - | - | T | G | A | - | - | A | A | - | C | - | G | - | T | - | G | - | - | - | OQ978582<br>OR073399<br>OQ978583 | EPI_ISL_14599870 | Ecuador | Homo sapiens | 2022-10-08 | Nasopharyngeal swab |
| USFQ12 | Omicron BA.2 | T | - | - | T | G | A | - | - | A | A | - | C | - | G | - | T | - | G | - | - | - | OQ978221<br>OR054012<br>OQ978220 | EPI_ISL_14208873 | Ecuador | Homo sapiens | 2022-07-14 | Nasopharyngeal swab |
| USFQ14 | Omicron BA.3 | - | - | - | T | - | A | - | - | A | A | - | C | - | G | - | T | - | G | - | - | - | OR073400<br>OQ978589 | EPI_ISL_13102254 | Ecuador | Homo sapiens | 2022-05-29 | Nasopharyngeal swab |
| USFQ11 | Omicron BA.4 | T | - | - | T | G | A | - | G | A | A | - | C | G | - | - | T | - | G | - | - | T | OR083677<br>OQ978586<br>OR073398<br>OR073397<br>OR141940 | EPI_ISL_14289590 | Ecuador | Homo sapiens | 2022-07-28 | Nasopharyngeal swab |
| USFQ13 | Omicron BA.4 | T | - | - | T | G | A | - | G | A | A | - | C | G | - | - | T | - | G | - | - | T | OQ978588<br>OR083678<br>OQ978587<br>OR054009<br>OR054008 | EPI_ISL_14599853 | Ecuador | Homo sapiens | 2022-08-10 | Nasopharyngeal swab |
| USFQ10 | Omicron BA.5 | T | - | - | T | G | A | - | G | A | A | - | C | G | - | - | T | - | G | - | T | - | OQ978585<br>OR083682<br>OQ978584<br>OR073396<br>OR073395 | EPI_ISL_14599895 | Ecuador | Homo sapiens | 2022-07-28 | Nasopharyngeal swab |
| USFQ15 | Omicron BA.5 | T | - | - | T | G | A | - | G | A | A | - | C | G | - | - | T | - | G | - | T | - | OQ978591<br>OR083681<br>OQ978590<br>OR054011<br>OR054010 | EPI_ISL_14599869 | Ecuador | Homo sapiens | 2022-08-11 | Nasopharyngeal swab |
| 28869 | Omicron BA.5 | T | - | - | T | G | A | - | G | A | A | - | C | G | - | - | T | - | G | - | T | - | OQ978644<br>OQ978643<br>OQ978641<br>OQ978642<br>OQ978645 | N/D | Ecuador | Homo sapiens | 2022-06-09 | Nasopharyngeal swab |
| 28875 | Omicron BA.5 | T | - | - | T | G | A | - | G | A | A | - | C | G | - | - | T | - | G | - | T | - | OR077469<br>OR083676<br>OR143349<br>OQ978646 | EPI_ISL_17708353 | Ecuador | Homo sapiens | 2022-06-14 | Nasopharyngeal swab |

Table S1-Diagnostic Mutations of samples used for validation, the hyphen means no occurrence of mutation with respect to the Wuhan reference sequence (NC_045512.2)
